# Improving Iodine Status in Lactating Women: What Works?

**DOI:** 10.1007/s13668-022-00427-y

**Published:** 2022-07-22

**Authors:** Louise Brough

**Affiliations:** School of Food and Advanced Technology, Massey University, Palmerston North, 4442 New Zealand

**Keywords:** Iodine deficiency, Universal salt iodization, Thyroid function, Breastfeeding, Infancy, Iodine supplementation

## Abstract

**Purpose of Review:**

Iodine deficiency is a global concern, and in recent years, there has been a significant improvement in the number of countries identified as being iodine-sufficient. This review considers the best strategies to ensure iodine sufficiency among breastfeeding women and their infants.

**Recent Findings:**

Fortification strategies to improve iodine intake have been adequate for school-age children (SAC); however, often, iodine deficiency remains for breastfeeding women and their infants. Daily supplementation with iodine is not an ideal strategy to overcome deficiency.

**Summary:**

Countries defined as iodine-sufficient, but where pregnant and breastfeeding women have inadequate intake, should consider increasing salt iodine concentration, such that the median urinary iodine concentration of SAC can be up to 299 µg/L. This will ensure adequate intake for mothers and infants, without SAC being at risk thyroid dysfunction. Consensus is required for thresholds for iodine adequacy for breastfeeding women and infants.

## Introduction

Adequate intake of dietary iodine is essential for the production of thyroid hormones, thyroxine (T4), and triiodothyronine (T3), required for the control of metabolic processes and growth and development, especially of the brain and central nervous system up to 3 years of age [1]. Iodine deficiency throughout the lifecycle can cause a number of detrimental health effects termed iodine deficiency disorders (IDD), the most common being goiter characterized by enlargement of the thyroid gland and hypothyroidism [2]. Iodine deficiency is of particular concern for pregnant and breastfeeding women and infants because of the essential role of thyroid hormones in fetal and infant brain development [3, 4]. Exposure to severe iodine deficiency in utero has the most damaging effects and can result in cretinism, characterized by serve mental impairment and negative impacts on growth and development [2]. However, even mild-to-moderate iodine deficiency during early life has the potential to impair cognitive development [4], which could have potential negative impacts for the rest of that child’s life and a cumulative impact for society.

Iodine deficiency affects many people throughout the globe, affecting low-, middle-, and high-income countries. The Iodine Global Network is a non-government, non-profit organization with the aim to reduce iodine deficiency globally. The most recent Iodine Global Network scorecard (2021) indicates a significant increase in recent years in countries categorized as iodine-sufficient, from 67 countries in 2003 to 118 in 2021. Nonetheless, 21 countries are still iodine-deficient, and 13 have excessive intakes [5, 6]. However, even some countries which are currently considered iodine-sufficient report iodine deficiency among pregnant women [7].

The aim of this narrative review is to discuss dietary iodine recommendations, the difficulties in assessing iodine status, and the adverse effects of deficient or excess iodine intakes and evaluate supplementation versus salt fortification for improving iodine status among breastfeeding women and their infants.

## Dietary Iodine Recommendation for Breastfeeding Women and Infants

Iodine requirements are high in breastfeeding as iodine is transferred to the infant via breast milk as well as being required for maternal thyroid function. Expression of the sodium iodide symporter increases in breast tissue during late pregnancy and lactation [8]. There is variation in dietary recommendations for iodine among lactating women and infants (Table 1). The National Academy of Medicine (formerly the Institute of Medicine) set the estimated average requirement (EAR) and recommended dietary allowance (RDA) at 209 and 290 µg/day during breastfeeding, compared to 95 and 150 µg/day for non-pregnant, non-breastfeeding women [9]. The WHO suggests the recommended nutrient intake (RNI) of 150 µg/day for women of childbearing age increasing to 250 µg/day during lactation [10].

**Table 1:** Dietary recommendations for iodine for lactating women and infants

	US National Academy of Medicine ^a^			WHO ^b^	
Age and stage	Estimated average requirement (EAR) (µg/day)	Recommended dietary allowance (RDA) (µg/day)	Tolerable upper level of intake (UL) (µg/day)	Recommended nutrient intake (µg/day)	Level intake beyond which no added health benefit can be expected (µg/day)
Women	95	150	900 [14–18 years] 1100 (≥ 19 years)	150	> 500
Lactating women	209	290	900 (14–18 years) 1100 (≥ 19 years)	250	> 500
	Adequate intake µg/day			Lower level of intake µg/day	
0–6 months	110		Insufficient data to determine	90 (< 2 years)	> 180 (< 2 years)
7–12 months	130				

^a^National Academy of Medicine [9]

^b^World Health Organization [10]

For infants, the National Academy of Medicine recommends an adequate intake (AI) of 110 and 130 µg/day for infants aged 0–6 months and 7–12 months, respectively [9]. The WHO recommends a lower level of intake of iodine intake of 90 µg/day for children less than 2 years old [10].

## Challenges of Defining Iodine Status

Assessing iodine status is problematic as there is no biomarker which can determine iodine status in an individual. The WHO recommends determining iodine concentrations in spot samples among a representative sample (*n* = 100–500) and comparison of median urine iodine concentration (mUIC) with recommended population thresholds [11, 12]. The use of school-age children (SAC, mUIC 100–199 µg/L) is advocated as a proxy for population status, as the epidemiological evidence is strongest for this age group [5, 11]. There is currently no agreed mUIC for adult populations, and the reference value for SAC is often incorrectly used leading to a potential overestimation of deficiency. Children usually have a lower urine volume than adults, so despite a lower iodine intake, their UIC will be higher. An mUIC threshold of below 60–70 µg/L is suggested as an appropriate indicator of deficiency among an adult population [13]. One-spot urine sample cannot be used to determine individual status as there is too much individual variation in daily intake, UIC can also be affected by hydration status and circadian rhythm [14, 15]. Pooled urine collected over 24 h can overcome some of these issues but not the considerable day-to-day variation in intake [16]. At least 10 to 12 samples would be required from an individual to determine individual status [12, 17]. Robust laboratory testing is essential to ensure validity and reproducibility using certified reference materials [16].

For lactating women and infants aged younger than 2 years, the mUIC threshold is currently set at 100 µg/L (Table 2), but there is no robust evidence for this recommendation [10]. The use of UIC to determine status is based on the principle that a constant proportion of iodine is excreted via urine (about 90%) [13]. Dold et al. investigated iodine status among breastfeeding women in a four-country study (*n* = 866) of three iodine-sufficient countries (China, Philippines, and Croatia) and one iodine-deficient country (Morocco) [18]. The researchers found that in iodine-sufficient countries, as iodine intake decreased, iodine was increasingly partitioned into breast milk, and hence, the fraction of iodine in urine decreased, whereas in the iodine-deficient country, the proportion in of iodine in breast milk remained constant. A small New Zealand study (*n* = 87) found a similar variance in partitioning of iodine between breast milk and urine dependent upon status [19]. Further, a recent Iranian study found that although maternal UIC and BMIC were associated with neonatal UIC, BMIC was the stronger predictor of neonatal UIC [20]. This suggests that UIC may not be an optimal measure status for breastfeeding women.

**Table 2:** Suggested population cutoffs for iodine adequacy for breastfeeding women and infants

Age and stage	Median urinary iodine concentration (mUIC, µg/L)	Author	Median breast milk iodine concentration BMIC (µg/L)	Author
Lactating women	≥ 100	Andersson et al. [10]	≥ 178	Dold et al. [18]
			100–200	Andersson and Braegger [3]
Infants (< 2 years)	≥ 100	Andersson et al. [10]		
	≥ 125	Dold et al. [22]		
	180–225	Delange (21)		

Delange [21] suggested a higher mUIC range for adequacy for infants of 180 to 225 µg/day based on a daily urine volume of 0.4–0.5 L/day and the WHO recommended intake of 90 µg/day of iodine. Using data from an iodine balance study, Dold et al. recommended a slightly higher threshold for adequacy of 125 µg/L equivalent to an EAR of 72 µg/day and RDA of 80 µg/day [22]. Collecting urine from infants is more difficult than for adults which limits the use of this method.

Breast milk iodine concentration is also a potential measure of maternal status and a proxy measure of intake for breastfed infants (Table 2). However, there is no current consensus on what constitutes adequacy with suggestions ranging between 75 and 100 µg/L, based on varying dietary recommendations [23–25]. Corresponding to recent data from three iodine-sufficient countries, Dold et al. suggest a reference range of 60–465 µg/kg (62–384 µg/L) iodine in breast milk for exclusively breastfeeding women and a target median range of 171 µg/kg (178 µg/L) [18]. However, due to the variation in partitioning of iodine between urine and breast milk dependent upon status, it would be best to measure both UIC and BMIC. Andersson and Braegger reviewed several studies and suggest an optimal range of median BMIC from 100 to 200 µg/L [3], but there is currently no agreed threshold.

Homeostatic regulation of thyroid hormones means they are often not indicative of iodine status; even in deficient populations, thyroid hormone concentrations can be within normal reference ranges [14]. Although thyroid-stimulating hormone (TSH) concentrations reflect status in new-born infants, the measure cannot be used in other population groups [26]. Postpartum thyroiditis (PPT) occurs in the first-year postpartum affecting around 5% of women. PPT is an autoimmune disorder characterized by thyroid antibodies (TPOAb and TgAb) which can manifest as hypothyroidism, hyperthyroidism, or both, though this is usually transient and returns to normal after 1 year [27]. This again complicates the use of thyroid hormones as markers of iodine status during lactation.

Thyroid volume has also been used to identify goiter by palpation of the neck or using ultrasound. However, palpation has poor sensitivity and specificity in areas with mild iodine deficiency [28]. Although ultrasound is more reliable, it is still a subjective measure and prone to intraobserver error [14]. There are also no reference ranges available for lactating women or infants. Further, there is a considerable lag time between the thyroid returning to normal size after iodine intake becomes adequate of months, even years [25].

Thyroglobulin (Tg) is a protein produced by the thyroid gland which is raised with both deficient and excessive iodine intakes [29]. Thyroglobulin can be measured in serum or dried blood spot samples [30–32]. The use of thyroglobulin as a biomarker is complicated by the presence of anti-thyroglobulin antibodies (TgAb), found in 3–13% of adults, which could lead to an underestimation of Tg [29]. There are currently no reference ranges for Tg for lactating women, and the presence of TgAb in PPT could affect its usefulness as a marker. Children are unlikely to have TgAb, and a threshold for deficiency for Tg (population median < 13 µg/L) has been suggested for children aged 6–12 years, based on a standardized method [33]. Recent research suggests thyroglobulin is also a sensitive biomarker in infants aged 6–24 months as concentrations were associated with iodine status, although not thyroid dysfunction [34]. Tg concentrations are raised in the neonate, due to raised TSH in the first few days of life, and then reduce with age [35]. However, there are currently no agreed reference ranges for infant aged less than 6 months.

Thyroglobulin more rapidly reflects changes to dietary iodine intakes than thyroid volume [36]; however, there is still a lag time which was clearly demonstrated in recent studies in New Zealand. In 2002, a nationwide study of SAC found median UIC, and serum Tg concentrations were 68 µg/L and 12.9 µg/L, respectively, which were indicative of iodine deficiency [37]. In 2009, the mandatory fortification of bread with iodized salt was introduced. A study of SAC in 2010/2011 found a median UIC of 113 µg/L indicating adequacy, and the median serum Tg concentration was 10.8 µg/L [38]. A follow-up study of SAC in 2015 found a similar UIC of 116 µg/L; however, this time, median Tg concentration had reduced further to 8.7 µg/L [39]. These studies suggest that the adequacy of intake is reflected more quickly in the UIC than in Tg, as a longer time is required for the thyroid to achieve optimal function.

## Consequences of Iodine Deficiency for Breastfeeding Women and Their Infants

In mild iodine deficiency, both serum Tg and thyroid size increase, although there may be changes to TSH, T3, and T4, these are usually not detected as they are within healthy reference ranges [40]. With moderate iodine deficiency, there is a slight increase in TSH, although T4 remains unchanged, and numerous people will develop subclinical hypothyroidism [40]. However, with severe iodine deficiency, TSH is raised, and T3 is preferentially secreted by the thyroid, to spare iodine [41]. Thus, T3 increases slightly or stays the same, and T4 decreases, resulting in an increase in the T3/T4 ratio. Many individuals will develop goiter and overt hypothyroidism [40].

The most damaging effects of iodine deficiency are observed when severe iodine deficiency occurs during pregnancy, resulting in serve mental impairment and adverse impacts on growth and development for the offspring, known as cretinism [2]. Mild-to-moderate iodine deficiency during pregnancy has also been shown to adversely affect thyroid hormones [42], and observational studies have suggested that mild-to-moderate maternal iodine deficiency can have adverse effects on neurodevelopment in children [43, 44]. A meta-analyses of Chinese studies suggested that children living in areas with iodine deficiency have a reduction of IQ of around 12 points compared iodine-sufficient children [45]. However, it is difficult to tease out the direct effect of iodine deficiency during infancy on neurological development. It is challenging to determine whether adverse consequences are solely due to in utero deficiency or if intake during the first few years of life has any further effect.

Iodine status during pregnancy is thought to affect fetal growth through the action of thyroid hormone on growth hormone and insulin-like growth hormone; iodine supplementation of pregnant women with severe iodine deficiency increased mean birth weight by 200 g [46]. However, the role of iodine adequacy in infant growth is also currently unclear; research has shown iodine repletion in children increases insulin-like growth factor but not growth [46]. One study investigating iodine supplementation in preterm infants showed no effect on growth [47], but adequately, powered studies of the effect of iodine on postnatal growth in term infants are needed [46].

## Consequences of Iodine Excess

A high dose of iodine results in an acute reduction of thyroid hormone synthesis, known as the Wolff–Chaikoff effect [48]. If the high iodine concentration continues, the thyroid adapts and escapes from this effect by downregulating the expression of the sodium iodide symporter which reduces iodine transport into the thyroid and ultimately resumes thyroid hormone production. An inability to escape the Wolf–Chaikoff effect results in iodine-induced hypothyroidism; this has been reported in neonates receiving high doses of iodine [48]. High maternal iodine intake and high breast milk iodine concentrations may result in hypothyroidism in breastfed infants; this is usually transient, but there is the risk of permanent thyroid dysfunction [3].

Conversely, an acute dose of iodine can result in hyperthyroidism, which can be transient and is more common with iodine deficiency and diffuse nodular goiter. Excess iodine intake may also result in thyroid autoimmunity [49]; however, thyroid antibodies are rare in children, and there is little data about the effect of excess iodine among breastfeeding women.

## Treatment of Iodine Deficiency: Universal Salt Iodization Versus Supplementation?

The WHO currently recommends that universal salt iodization is the most effective method to ensure an adequate iodine intake throughout the whole population [10]. In countries or regions where less than 90% of households consume iodized salt, breastfeeding women should receive either a daily supplement to ensure their intake reaches the RNI of 250 µg/day or a single oral dose of iodized oil containing 400 mg iodine annually [10]. Many countries recommend postnatal supplementation with 150 µg iodine daily [3].

In many countries with USI, fortification is usually sufficient for SAC and adults; however, in some countries’ iodine intake is still inadequate among breastfeeding women. In a systematic review of research studies investigating mUIC among breastfeeding women up to 2013, Nazeri et al. found that among 21 studies in countries with mandatory fortification programs, 8 countries (Australia, India, Denmark, Mali, New Zealand, Slovakia, Sudan, and Turkey) had areas with mUIC below 100 µg/L [50]; this could suggest deficiency although the partitioning of iodine dependent on mothers status could mean that not all these areas are deficient. In a later meta-analysis of BMIC and infant UIC up to 2016, Nazeri et al. found there was no significant difference in mean BMIC in mature breast milk between iodine-sufficient and iodine-deficient countries (71.5 μg/L and 28.0 μg/L, respectively) [51]. However, the mean BMIC is below the 100–200 µg/L advocated as being sufficient by Andersson and Braegger [3], and of the 21 studies in sufficient countries, six countries (Chile, China, Iran, Slovakia, Switzerland, and USA) had a mean BMIC below 100 µg/L. A recent cross-sectional multicenter study in three countries (China, Philippines, and Croatia) with mandatory USI demonstrated that salt iodization of around 25 mg/kg was required to ensure adequate iodine intake of the whole population, including breastfeeding women and their infants [52].

In Australia and New Zealand, salt iodization is voluntary; however, the addition of iodized salt to bread was mandated in 2009 to ensure sufficiency for the majority of the population [53]. This was predicted to be inadequate for pregnant and breastfeeding women; thus, an iodine supplement (150 µg/day) is also recommended for these women [54, 55]. Although these initiatives have improved iodine status for much of the population, this improvement is not universal; some pregnant and breastfeeding women and infants still have inadequate status [56–61]. However, it should be noted there is no nationally representative data for pregnant and breastfeeding women, and information about these groups is from smaller studies.

Using daily iodine supplementation to address deficiency has limitations as there are several barriers to supplement use and uptake is often less than optimal [62]. In small New Zealand studies of educated women, iodine supplement use ranged from 6 to 40% among breastfeeding women and reduced with infant age [19]; deficiency was evident among non-users of supplements. Iodine supplement use is also lower among mothers who are younger and less educated and among some ethnic groups (Māori or Pacifica in New Zealand and Indigenous Australians) [63, 64]. The costs associated with supplement use and inadequate access to health services by some ethic groups are possible reasons for lack of supplement use.

The effect of providing a single large dose of iodized oil orally to either breastfeeding women or their infants (median age 2 weeks) was investigated in Morocco, a country with severe iodine deficiency [65]. Either lactating women were given 400 mg iodine and the infant a placebo or infants were given 100 mg and mothers a placebo. Iodine status was higher in the infants when the mother received the treatment rather than it being given directly to the infant. This could be due to neonates having lower thyroidal iodine than adults and a higher iodine turnover [25]. BMIC was slightly higher in the mothers who received treatment compared to placebo at 3, 6, and 9 months. However, both BMIC and infant mUIC were still lower than that reported in iodine-replete countries [18, 52].

Robust research suggests that even mild iodine deficiency during pregnancy can adversely affect cognitive function [43, 44]. However, numerous international studies investigating iodine supplementation during pregnancy have seen no demonstrable improvement in either maternal thyroid function or neurodevelopment. Even the gold standard of research, randomized controlled trials, has failed to any show a clear benefit [66]. One possible cause for this could be that if iodine supplementation is provided to an iodine-deficient women in early pregnancy, the sudden increase in iodine availability can result in a transient inhibition of thyroid hormone production and release, in a manner similar to the Wolff–Chaikoff effect, which could have adverse effects on the developing fetus [42]. Thus, it would seem that ensuring adequate iodine intake prior to pregnancy until the end of lactation would ensure optimal health outcomes for mother and infants. It is more difficult for supplementation programs to obtain a wide reach compared to fortification of the food supply.

The WHO previously advocated that for population to have an adequate iodine intake, the mUIC of SAC should be in the range 100–199 µg/L, the range 200–299 mUIC µg/L may increase the risk of hypothyroidism in some groups, and mUIC > 300 µg/L was classed as excessive with risks of adverse health consequences for the population [11]. This was used as the basis for some countries’ fortification strategies, to ensure the mUIC of SAC falls within in the range 100–199 µg/L. However, research has demonstrated that no thyroid dysfunction is seen among SAC when the mUIC is in the range 100 up to 299 µg/L [33]. In countries with mandatory fortification where iodine intake is adequate for the majority of the population, mothers and infants have suboptimal intake consideration that should be given to increasing the iodine concentration in iodized salt to a level which is adequate for mothers and infants but not excessive for other children. This will require dietary careful dietary modeling to ensure children are not exposed to toxic intakes.

## Conclusion

It is essential that breastfeeding women consume adequate iodine to ensure optimal thyroid function for themselves and their breastfed infants. It is difficult to measure individual iodine status, and population measures are usually used. mUIC is the preferred population measure, and this is suitable for infants, although the threshold for adequacy is currently undecided. However, for breastfeeding women, mUIC is not always a reliable indicator of iodine status due to variable partitioning of iodine between breast milk and urine; thus, both BMIC and mUIC should be determined. Universally agreed thresholds for adequacy for both mothers and infants need to be established. Ensuring adequate iodine throughout the population is a balancing act, ensuring pregnant and breastfeeding women and their infants have adequate intake and safeguarding other children from excessive levels. USI is consistently recommended as the best strategy to achieve population adequacy, and this approach should be implemented in iodine-deficient countries if possible. However, even among countries which are classified as iodine-sufficient, pregnant and breastfeeding women and their infants do not always have adequate intake. Supplementation is often used to address iodine insufficiency among pregnant and breastfeeding women, but there are barriers to supplement the use and uptake which is often less than ideal. In countries with mandatory fortification currently defined as iodine-sufficient, where pregnant and breastfeeding women have inadequate intake, policy makers should consider the broad mUIC range which is adequate for optimal thyroid function among SAC (100–299 µg/L). Careful increases in iodine concentrations in salt, based on robust dietary modeling, could ensure adequate intake for mothers and their infants without SAC being subjected to excessive intake and thyroid dysfunction.
